# Quality and Misinformation About Health Conditions in Online Peer Support Groups: Scoping Review

**DOI:** 10.2196/71140

**Published:** 2025-05-16

**Authors:** Bethan M Treadgold, Neil S Coulson, John L Campbell, Jeffrey Lambert, Emma Pitchforth

**Affiliations:** 1 Exeter Collaboration for Academic Primary Care Health and Community Sciences, Faculty of Medicine University of Exeter Exeter United Kingdom; 2 School of Medicine Faculty of Medicine & Health Sciences University of Nottingham Nottingham United Kingdom; 3 Centre for Motivation and Behaviour Change Department for Health University of Bath Bath United Kingdom

**Keywords:** quality, online, health, peer, online support group, forum, information, advice, approval, assessment, PRISMA

## Abstract

**Background:**

The use of health-related online peer support groups to support self-management of health issues has become increasingly popular. The quality of information and advice may have important implications for public health and for the utility of such groups. There is some evidence of variable quality of web-based health information, but the extent to which misinformation is a problem in online peer support groups is unclear.

**Objective:**

We aimed to gain insight into the quality of information and advice about health conditions in online peer support groups and to review the tools available for assessing the quality of such information.

**Methods:**

A scoping review was undertaken following the Joanna Briggs Institute scoping review methodology. We searched electronic databases (MEDLINE [Ovid], CINAHL, Web of Science, ASSIA, ProQuest Dissertation and Theses, and Google Scholar) for literature published before November 2023, as well as citations of included articles. Primary research studies, reviews, and gray literature that explored the quality of information and advice in online peer support groups were included. Title and abstracts were independently screened by 2 reviewers. Data were extracted and tabulated, and key findings were summarized narratively.

**Results:**

A total of 14 (0.45%) relevant articles, from 3136 articles identified, were included. Of these, 10 (71%) were primary research articles comprising diverse quality appraisal methodologies, and 4 (29%) were review articles. All articles had been published between 2014 and 2023. Across the literature, there was more evidence of poor quality information and misinformation than of good quality information and advice, particularly around long-term and life-threatening conditions. There were varying degrees of misinformation about non–life-threatening conditions and about mental health conditions. Misinformation about noncommunicable diseases was reported as particularly prevalent on Facebook. Fellow online peer support group users often played an active role in correcting misinformation by replying to false claims or providing correct information in subsequent posts. Quality appraisal tools were reported as being used by researchers and health care professionals in appraising the quality of information and advice, including established tools for the appraisal of health-related information (eg, DISCERN, HONcode criteria, and *Journal of the American Medical Association* benchmark criteria). No tools reported were specifically designed to appraise online peer support group content.

**Conclusions:**

While there is good quality information and advice exchanged between users in online peer support groups, our findings show that misinformation is a problem, which is a matter of public health concern. Confidence in the quality of information shared may determine the utility of online peer support groups for patients and health care professionals. Our review suggests that clinical and academic experts in health conditions could play a valuable role in ensuring the quality of content. Several quality appraisal tools are available to support such an initiative.

## Introduction

### Background

In recent years, there has been a rapid increase in people’s use of web-based resources aimed at meeting their health information needs [1,2]. Seeking health information and advice from the internet has been described as the interaction of an individual (eg, patient, caregiver, or professional) with or through an electronic device or communication technology to access or to transmit health information [3]. A large amount of web-based health information is generated by health care providers; pharmaceutical companies; and other public, private, and charitable health organizations by virtue of websites for the public. At the same time, an increasing volume of web-based health content is being generated by individuals living with or caring for others with a health condition, via online peer support groups [4-10].

Online peer support groups, otherwise named online support communities, are virtual spaces where individuals exchange health-related information (ie, factual knowledge that is not based on a person’s opinion); advice (ie, a recommendation for action based on someone’s experience); and peer support (ie, sharing personal experiences and emotional, social, or practical support with one another) often through asynchronous, text-based computer-mediated communication platforms (eg, discussion forums and social media sites) [4,10,11]. Exchanging health-related information and advice in online peer support groups has been found to empower people living with or caring for others with health conditions in learning new methods for managing their conditions, in feeling that their experiences are validated, in reducing a sense of loneliness, and in improving their navigation of health care services [7-9,12-14]. Online peer support groups have been described as a health care resource that is complementary to formal health care, with patients feeling as equal contributors in the patient–health care provider relationship [10]. Interactions through online peer support groups about health can also lead to the identification of health-related problems that health care professionals have not yet considered, and sharing health-related experiences through such groups has led to campaigns to improve health care [15].

While online peer support groups have become increasingly popular, there is growing concern from health care professionals and researchers regarding the accuracy and safety of some health information on the internet [16-19]. The concept of information quality has multiple dimensions, centered on trustworthiness and scientific correctness [20]. Increasing attention has been given to exploring the quality of health-related information and advice exchanged in online peer support groups, particularly around the medical accuracy of content or, conversely, the spread of misinformation [21-23]. Health-related misinformation has been defined as a health-related claim of a fact that is false due to a lack of scientific evidence [24] and is usually attributed to misconceptions. Exposure to misinformation in online peer support groups and following false information and advice can detrimentally impact health outcomes [25-31]. Several incidents of public health concerns arose out of misinformation spreading across social web-based platforms during the COVID-19 pandemic, including hospitalization and casualties [32]. Poor quality information often reproduces within discussion threads in online peer support groups, where like-minded people come together and similar topics circulate (the “echo chamber effect”) [30]. People with lower levels of health literacy may be particularly vulnerable to the detrimental impacts of misinformation [29,31].

### Rationale for the Study

With the rapid spread of misinformation in online peer support groups posing significant threats to public health, a comprehensive scoping review of the quality of information and advice and the methods used in quality appraisals is needed. Knowledge about the quality of information shared in online peer support groups is directly relevant for patients, carers, and health care professionals in supporting the management of health conditions. In addition, an understanding of the quality appraisal methods is important, as they serve as potential moderators of the quality of information and advice provided.

### Review Objective

The objective of this scoping review was to map and summarize evidence about the quality of information and advice in online peer support groups across a range of health conditions and to review the available tools used to assess the quality of such information and advice.

### Review Questions

The following were our research questions (RQs):

- RQ 1: What is known about the quality of information and advice exchanged within online peer support groups about health conditions?
  RQ 1.1: To what extent is misinformation about health conditions a problem within online peer support groups?
  RQ 1.2: How have researchers assessed the quality of information and advice exchanged in online peer support groups about health conditions?

## Methods

### Overview

Scoping reviews aim to identify and map the key concepts within a research area regarding its nature, features, and volume. Such reviews often address a broad RQ and include a variety of study designs [33,34]. This scoping review was conducted in accordance with the Joanna Briggs Institute methodology for scoping reviews [35-37] and followed the PRISMA-ScR (Preferred Reporting Items for Systematic Reviews and Meta-Analyses Extension for Scoping Reviews) checklist for reporting [38]. The review protocol was registered in January 2024 on the Open Science Framework Registries [39]. The PCC (Population, Concept, and Context) framework [37] was followed as a guide to construct the review objectives, search strategy, and eligibility criteria. Covidence systematic review software was used for all review stages [40].

### Search Strategy and Procedure

The principal reviewer (BMT) and second reviewer (EP) developed the search strategy, with assistance from a university research librarian. The search strategy aimed to identify published and unpublished literature and focused on three central concepts: (1) online support groups, (2) quality assessment, and (3) health-related information and advice. An initial limited search was conducted in the Web of Science and MEDLINE (Ovid) databases to identify the literature on this topic and keywords referring to the 3 central concepts of the search strategy (Textbox 1). Records identified through this initial search were reviewed to identify relevant literature, and text words contained in the titles, abstracts, and keywords of relevant literature were used to identify additional keywords to develop the final search strategy (Multimedia Appendix 1). An adapted search strategy was developed for each database.

**Preliminary keywords referring to 3 central concepts (online support groups, quality assessment, and health-related information and advice) used to develop the final search strategy for this scoping review.**

**Online support group search terms**

- online support group
- online support groups
- online community
- online communities
- discussion forum
- discussion forums
- social media
- social networking sites

**Quality assessment search terms**

- quality assessment
- quality
- assessment medical accuracy
- accuracy
- appraisal

**Health-related information search terms**

- information
- advice
- health

We searched electronic databases (MEDLINE [Ovid], CINAHL, Web of Science, and Applied Social Sciences Index and Abstracts) and sources of unpublished and gray literature (Google Scholar and ProQuest Dissertations and Theses) from inception to November 2023. Google Scholar collates results from across the internet and does not have internal functions for an advanced search as do the other included databases. Previous research indicated that most gray literature begins to appear after approximately 20 to 30 pages in Google Scholar results; therefore, the first 100 results from page 20 onward were screened [41]. The reference lists of included review articles and citations of all included articles were hand-searched for additional relevant literature. Where full texts were not available, authors were contacted to request a copy.

### Eligibility Criteria

Inclusion and exclusion criteria reflected the focus of this scoping review on the quality of information and advice about health conditions in online peer support groups (Textbox 2).

**Eligibility criteria for identifying the literature exploring the quality of information and advice about health conditions in online peer support groups.**

**Inclusion criteria**

- Population
  Synchronous or asynchronous text-based online peer support groups, where individuals can exchange information, advice, and support about health conditions within an online community or group or through a discussion thread about a given topic (eg, Facebook support groups, Reddit, Twitter [rebranded as X], Instagram, WhatsApp, Mumsnet, Health Unlocked, Patient, and charity-owned online peer support groups)
- Concept
  The quality of information and advice about health conditions in online peer support groups (eg, medical accuracy, comparison of information with evidence-based guidelines and relevant literature, readability and usability of content, and degree of misinformation)
  Quality assessment tools and questionnaires (eg, DISCERN tool, Health on the Net Code of Conduct, and the *Journal of the American Medical Association* benchmark) and their application to information and advice about health conditions in online peer support groups
- Context
  Primary peer-reviewed studies and reviews using quantitative, qualitative, or mixed methods design; dissertations and theses; and gray literature that explored the quality of information and advice about health conditions in online peer support groups
  References within included review articles and citations of included articles
  Studies from any geographic location, focusing on any type of health condition, and published at any time and in any language

**Exclusion criteria**

- Population
  Video-based social media sites, which do not involve joining an online peer community or group or the use of discussion threads (eg, YouTube and TikTok)
- Concept
  The quality of information and advice on websites that only provide static information that cannot be contributed to or discussed in an online peer support group format (eg, the National Health Service website, WebMD, National Institute of Health, Mayo Clinic, and health-related charity websites).
  The quality of information and advice that is from an online peer support group that is not focused on a particular health condition (eg, health promotion, dieting, and pregnancy).
  The quality of any health-related support services organized by health care professionals (ie, online support interventions conducted as part of a research trial).
  The quality of information and advice in non–online-based support groups (eg, in-person support groups).
- Context
  Conference abstracts and proceedings, commentaries, books, and editorials.

### Selection of Included Articles

All identified citations were collated and uploaded into the review software, and duplicates were removed. Two independent reviewers (BMT and EP) screened titles and abstracts against the inclusion criteria. In cases where the abstract signaled potential eligibility, the full article was retrieved and imported into Covidence, read, and assessed for inclusion. Reasons for exclusion of articles were recorded as full text. Any disagreements at each stage of the selection process were resolved through discussion.

### Data Extraction

Data were extracted by BMT using a data extraction tool developed by the wider review team (BMT, NSC, JLC, JL, and EP). The data extraction tool included predetermined study characteristics (ie, authors, year, country, research design methodology, and literature aims), conditions in focus of an online peer support group, key findings, details of quality assessment tools, and study limitations. After extracting data from each included article, BMT sent completed data extraction to a second reviewer (EP) for consensus. The extracted study characteristics were discussed before agreeing on the final extracted content to be analyzed and synthesized.

### Data Analysis

Data extracted from included literature were tabulated and summarized narratively. Given the diversity and heterogeneity of the literature concerning methodologies and findings, a narrative summary of findings was chosen to provide a comprehensive understanding of the literature. BMT uploaded the full texts of included articles into NVivo (Lumivero) qualitative data analysis software [42] and coded their content according to the 3 RQs of this scoping review. Codes were analyzed descriptively and discussed with additional reviewers (EP and NSC). Final summaries of each RQ were developed with the wider review team (BMT, NSC, JLC, JL, and EP).

## Results

### Included Articles

**Figure 1:**
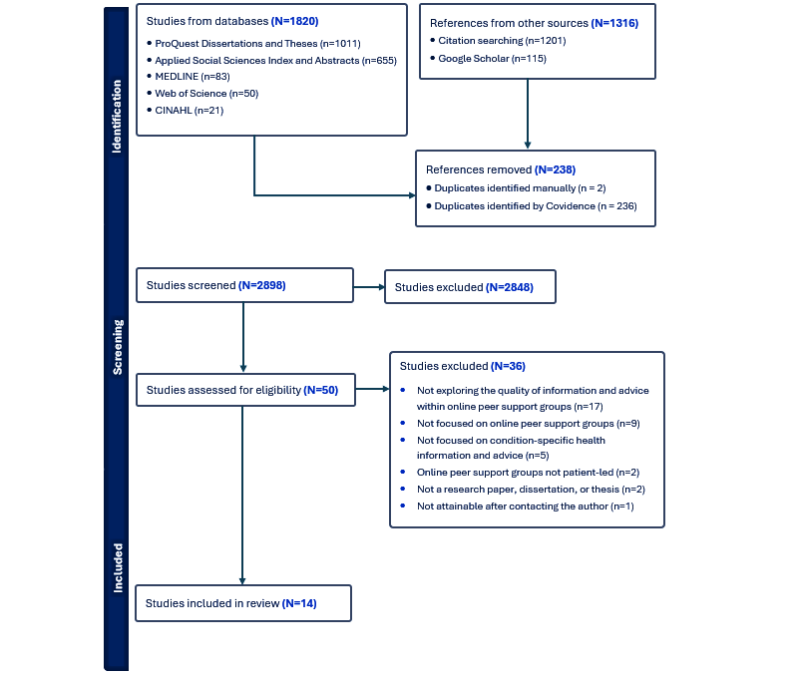
PRISMA flow diagram detailing the inclusion process for this scoping review on the quality of information and advice about health conditions in online peer support groups.

The search strategy yielded 3136 results, of which 238 (7.59%) were duplicates. From the 2898 titles and abstracts reviewed, 2848 (98.27%) did not meet the inclusion criteria. The full texts of the remaining 50 (n=2898, 1.72%) articles were assessed, and a further 36 (72%) were excluded as they did not meet the inclusion criteria (n=35, 70%) or were not attainable after contacting the author (n=1, 2%). Overall, there were 14 (28%) articles included in our review. Figure 1 presents a PRISMA (Preferred Reporting Items for Systematic Reviews and Meta-Analyses) [43] flow diagram detailing the inclusion process.

### Characteristics of Included Studies

Of the 14 included articles, 11 (79%) had been published since 2020. In total, 10 (71%) were primary research articles that reported a quality appraisal of information and advice about health conditions in online peer support groups and 4 (29%) were review articles. A total of 6 (43%) articles were published in the United States; 4 (29%) articles were published in the United Kingdom; and the remaining 4 (29%) articles were published in Ghana, Norway, Spain, and Korea. The included articles focused on a variety of health conditions, which sometimes fell into more than one category, including long-term conditions (n=14, 100%), acute conditions (n=7, 50%), physical health conditions (n=13, 93%), mental health conditions (n=3, 21%), and viral infections (n=6, 43%). Several web-based platforms were explored, including Facebook (n=8, 57%), X (formerly known as Twitter; n=7, 50%), and Reddit (n=6, 43%). Table 1 provides further details about the included articles.

**Table 1:** Characteristics of the 14 included articles of this scoping review exploring the quality of information and advice about health conditions in online peer support groups.

Study and year	Country	Aims	Research design methodology	Web-based platforms explored	Conditions in focus
Slick et al [44], 2023	United States	To assess the use of social media for medical information relevant to sickle cell disease and the accuracy of information provided for and by individuals with sickle cell disease	Extraction of discussion threads from Facebook groups and quality assessment	Facebook	Sickle cell disease
Afful-Dadzie et al [45], 2023	Ghana	To review research that focuses on health topics, users, and social media platforms that are raising health information quality concerns To review the suitability of existing criteria and instruments used in evaluating quality of social media health information	Literature review and content analysis	Twitter (rebranded as X), Facebook, Instagram, and WeChat	Cancer, dental or oral care, diabetes, heart condition or disease, sexual health, anorexia, alcohol, spinal disorder, eye, and inflammatory bowel disease
Ulep et al [46], 2022	United States	To synthesize existing research studies related to social media use concerning hearing loss, tinnitus, and vestibular disorders	Systematic review and descriptive analysis	Twitter, Facebook, Instagram, Reddit, and others unnamed	Hearing loss, tinnitus, and vestibular disorders
Skafle et al [47], 2022	Norway	To synthesize the existing research on misinformation about COVID-19 vaccines spread on social media platforms and its effects To gain insight and gather knowledge about whether misinformation about autism and COVID-19 vaccines is being spread on social media platforms	Rapid review and thematic analysis with narrative qualitative synthesis	Several social media platforms: WhatsApp, Facebook, Instagram, Twitter, and others	COVID-19
Pollack et al [48], 2022	United States	To quantify the presence of 4 types of obesity-related content on Reddit (misinformation, facts, stigma, and positivity) and identify psycholinguistic features that may be enriched within each one	Extraction of discussion threads from Reddit and using artificial intelligence (machine learning) to evaluate misinformation	Reddit	Obesity
Farnood et al [49], 2022	United Kingdom	To understand the types of information that people discuss when engaging with online health forums for heart failure and explore the quality of diagnostic advice provided	Extraction of discussion threads from online health forums and quality assessment	Public domain internet discussion forums (names not reported)	Heart failure
Towne et al [50], 2021	United States	To analyze the nature and accuracy of social media content related to endometriosis	Extraction of discussion threads from Facebook groups and content analysis	Facebook	Endometriosis
Suarez-Lledo and Alvarez-Galvez [51], 2021	Spain	To identify and compare the prevalence of health misinformation topics on social media platforms, with specific attention paid to the methodological quality of the studies and the diverse analytic techniques that are being implemented to address this public health concern	Systematic review (analysis method not reported)	Several social media platforms: Twitter, Facebook, MySpace, WhatsApp, and VK (formerly VKontakte)	Noncommunicable diseases, pandemics, and eating disorders
ElSherief et al [52], 2021	United States	To present machine learning and natural language analysis approaches to identify the characteristics and prevalence of web-based misinformation related to medication for opioid use disorder To inform future prevention, treatment, and response efforts.	Extraction of discussion threads from social media sites and online discussion forums and using artificial intelligence (natural language analysis approaches) to identify misinformation	Twitter, Reddit, and Drugs-Forum	Opioid use disorder
Du et al [53], 2021	United States	To develop and evaluate an intelligent automated protocol for identifying and classifying human papillomavirus vaccine misinformation on social media using machine learning–based methods	Extraction of discussion threads from Reddit and using artificial intelligence (machine learning) to evaluate misinformation	Reddit	Human papillomavirus infection
Jo et al [54], 2020	Korea	To investigate the trends of web-based health communication, analyze the focus of people’s anxiety in the early stages of COVID-19, and evaluate the appropriateness of web-based information	Extraction of questions and answers from an online discussion forum and using artificial intelligence (structural topic model) to evaluate content	The Naver questions and answers forum	COVID-19
Sepehripour et al [55], 2017	United Kingdom	To quantify the quality of information for patients with microtia on social media compared with other websites To compare physician and patient perspectives on the quality of patient information	Scoring of social media sites regarding quality	Facebook and Twitter	Microtia
Cole et al [56], 2016	United Kingdom	To provide an assessment of the quality and quality characteristics of information found in online discussion forums so that physicians, patients, and health care policy makers can better understand the online discussion forum environment and the information found there	Extraction of discussion threads from online discussion forum websites and quality assessment	Reddit, Mumsnet, and Patient	HIV infection, diabetes, and chicken pox
McGregor et al [57], 2014	United Kingdom	To assess the content and accuracy of material that patients with glaucoma can access on web-based social media platforms	Extraction of discussion threads from social media platforms and analysis using a combination of content analysis and simple thematic analysis	The international Glaucoma Association forum, Facebook, Twitter, Patient, and Care Opinion	Glaucoma

### Narrative Summary of Findings

#### RQ 1: What Is Known About the Quality of Information and Advice Exchanged Within Online Peer Support Groups About Health Conditions?

##### Overview

The included articles explored a variety of long-term, acute, physical, and mental health conditions (Multimedia Appendix 2 [44-57]). The quality of information and advice in online peer support groups varied according to the condition in focus and across web-based platforms. There were more reports of misinformation or mixed quality information about health conditions in online peer support groups than of good quality information.

##### Good Quality Information and Misinformation About Long-Term Conditions

In total, 10 (71%) included articles [44-46,48-51,55-57] explored the quality of information and advice about long-term physical health conditions in online peer support groups. Studies reported that the content about long-term life-threatening conditions, such as cancer and diabetes, raised the most substantial concerns regarding the quality of information, highlighting fake, misleading, or inaccurate information [45,51]. With regard to cancer, information about treatments and surgical procedures was particularly described as poor quality [45]. Conversely, 1 (7%) article [56] found that information about diabetes in online peer support groups was of reasonable quality and rarely contained inaccurate information. For heart failure, most of the information exchanged in online peer support groups has been described as of low quality and not aligned with evidence-based guidelines, with some described as potentially harmful [49]. Examples of harmful information and advice included instances where users confirmed diagnoses to other users that were incorrect and were not supported by existing evidence or guidelines [49]. Similarly, for sickle cell disease, most of the information and advice was rated as medically inaccurate, such as in relation to hydroxyurea medication, managing pain, and the use of natural supplements [44]. Non–life-threatening conditions, such as endometriosis [50], microtia [55] and glaucoma [57], were found to include little misinformation in comparison to the life-threatening conditions. Some cases of misinformation were reported in glaucoma-focused online peer support groups, which ranged from discussions about non–evidence-based treatments, such as carnosine eye drops and complementary therapies, to more dangerous advice around the use of medical marijuana [57]. For ear-related disorders, a greater amount of misinformation about tinnitus was reported than about hyperacusis, although the specific types of information exchanged were not reported [46].

##### Good Quality Information and Misinformation About Viruses

There were mixed findings about virus infections reported in 6 (43%) articles [47,49,51,53,54,56]. For COVID-19, information and advice about physical symptoms of COVID-19 were assessed as appropriate [54], whereas information about the COVID-19 vaccine was reported as misinformed [47,51], with misinformation about side effects such as infertility, chronic illness, changes in DNA, physical deformities, mental illness, and conspiracy theories [47]. Information and advice about human papillomavirus virus infection has been reported as being of mixed quality [51,53]. Examples of misinformation included the safety of the human papillomavirus virus vaccine, vaccine adverse effects, conspiracy theories about the government and pharmaceutical companies, and the prevalence of human papillomavirus virus infection [53]. With regard to chicken pox, some non–evidence-based discussions around vaccination and herbal remedies have been reported, but there was little evidence of poor quality or inaccurate information and advice [56]. Information and advice about HIV infection has been similarly reported to be of reasonably high quality with only a very small proportion considered to be factually incorrect or harmful [56].

##### Varying Degrees of Misinformation About Mental Health Conditions

In total, 3 articles (21%) [45,51,52] reported on the quality of information and advice in online peer support groups relating to mental health conditions. One study on opioid use [52] reported little misinformation despite anticipating the reproduction of a commonly held misunderstanding that medication-assisted treatment is simply replacing one drug with another. Another study [51] found a moderate amount of misinformation related to eating disorders, such as around normalizing eating disorders, changing eating habits, and using beauty as the final goal. Content related to eating disorders is frequently hidden or not evident to the public, as pro–eating disorder communities tend to use their own codes to reach specific audiences; thus, the prevalence of misinformation related to eating disorders in online peer support groups may be even greater than what is clearly visible [51].

##### The Quality of Information and Advice According to Web-Based Platforms

Across the included articles, 18 different web-based platforms were represented (Multimedia Appendix 3 [44-57]). The most commonly explored web-based platform was Facebook. One review reported that more studies until 2023 have explored Facebook than other web-based platforms, while other web-based platforms, such as WhatsApp, Instagram, WeChat, and country-specific web-based platforms are becoming more popular for seeking information and advice about health conditions [45]. Misinformation about noncommunicable diseases and treatments has been reported as particularly prevalent on Facebook [51]. Misinformation about vaccines and drugs has been reported as most prevalent on X [51,52]. On Reddit, misinformation about the human papillomavirus virus vaccine has been reported to cluster in “subreddits” [53], which are smaller communities that are themed around a given topic [48], although the degree of misinformation on Reddit has been reported to have decreased over time, possibly due to public health campaigns and an increase in internet verification skills among users [53]. In comparison to information and advice about health conditions in online peer support groups, the quality of the web-based platform itself was scarcely explored across the included articles.

#### RQ 1.1: To What Extent Is Misinformation About Health Conditions a Problem Within Online Peer Support Groups?

##### Overview

Health-related misinformation was a central focus across many of the included articles. Some included articles solely focused on misinformation and negated exploring good quality information. Attempts at addressing the spreading of misinformation in online peer support groups were also reported.

##### The Focus on Health-Related Misinformation in the Literature

Misinformation was reported to varying degrees in all 14 (N=14, 100%) articles, although misinformation received more attention than the general quality of information. In total, 5 (N=14, 36%) articles [47,48,51-53] specifically focused on variants of misinformation circulating in online peer support groups about various health conditions and did not consider the quality of information more broadly. As detailed earlier, misinformation in online peer support groups has been identified regarding long-term life-threatening conditions [44,45,49,51], viral infections [47,51,53,54], and mental health conditions [51,52]. Researchers have emphasized that in the event of a novel infectious disease outbreak where there is great uncertainty among the public, obtaining accurate information can be extremely difficult [54].

##### The Activities of Users in Minimizing Health-Related Misinformation

The degree of the problem of misinformation in online peer support groups can be reduced when fellow users address or correct false claims. Users have been observed to have corrected misinformation provided by other users through replying to false claims or through providing correct information in subsequent posts [45,53,56]. In addition, some users have also been observed to signpost fellow users to additional resources that are reliable and helpful, in response to misinformation [49]. Some online peer support groups such as Reddit operate a voting system on each discussion thread, whereby posts that contain good quality information and advice can be upvoted to the top of the discussion thread and lower quality posts can be demoted [56]. The need for health care professionals to take an active role in correcting misinformation in online peer support groups has been widely emphasized, a role that would involve dispelling false medical information that has the potential for spreading further on the web [45,47,51,52]. Organized web-based public health campaigns, composed of factual, evidence-based messages, have also been promoted to combat misinformation [51-53].

#### RQ 1.2: How Have Researchers Assessed the Quality of Information and Advice Exchanged in Online Peer Support Groups About Health Conditions?

##### Overview

There were various processes for appraising the medical accuracy of content regarding the types of professionals conducting the quality appraisal, the use of a quality appraisal tool to record the appraisal, and the consideration of quality indicators that might be used when appraising content.

##### Involvement of Health Care Professionals in Quality Appraising Information and Advice

In total, 9 (N=14, 64%) articles [44,48-50,54-57] reported involving academic or health care professionals in quality appraisal. In some cases, discussions in online peer support groups were appraised by researchers or clinical academics researching the conditions in focus [48-50,53], by secondary care health care professionals who were specialists or consultants in the conditions [44,55-57], by general practitioners [54,56], and by patients and carers with lived experiences of managing the conditions in focus [56].

##### Comparison of Information and Advice With Medical Guidelines

Evidence-based guidelines relevant to the condition were often used in determining the quality of information and advice exchanged [44,48-50]. Guidelines mentioned included the Scottish Intercollegiate Guidelines Network, National Institute for Health and Care Excellence, American Board of Obesity, and National Institute of Health–National Heart, Lung, and Blood Institute. Current evidence in peer-reviewed scientific journals [50] and information provided on trusted charity websites dedicated to the condition in focus [49] were also used as a guide for quality appraising content.

##### Use of Quality Appraisal Tools for Recording the Assessment

In total, 5 (36%) articles reported using specific quality appraisal tools [47,54-57]. The DISCERN instrument [58], the Health on the Net Code of Conduct (HONcode) [59,60], and the *Journal of the American Medical Association* (JAMA) benchmark [61] (Table 2) have been used in information quality evaluation across multiple social media and online peer support group platforms [45]. None of these tools were specifically designed to appraise online peer support group content, although they were still reported as being used for this purpose by health care professionals and patient users investigating the quality of such content. The DISCERN instrument, which contains 16 items, focuses on rating the quality of health information written by consumers about treatment choices [58], not necessarily web-based information or specifically for the assessment of information and advice in online peer support groups. The HONcode criteria contain 8 principles that are used as a guide in assessing the reliability and usefulness of medical information on health-related websites [59,60]. In addition, the HONcode criteria have also been used to appraise content in online peer support groups [45,46], despite being devised for traditional health information websites. Similarly, the JAMA benchmark [61] contains 4 core standards that have been used [45] as a guide in assessing health information on websites. One article reported developing their own tool specifically for the purpose of their research study instead of using an official quality appraisal tool [56]. Some quality appraisal studies reported combining 2 or more of the aforementioned quality appraisal tools to assess information and advice in online peer support groups [45]; however, the rationale behind this was not explained.

**Table 2:** Summary of the DISCERN, Health on the Net Code of Conduct (HONcode), and Journal of the American Medical Association (JAMA) benchmark quality appraisal tools.

Quality appraisal tool	Aim of tool	Criteria or items
DISCERN [58]	To help users of consumer health information judge the quality of written information about treatment choices	Are the aims clear? Does it achieve its aims? Is it relevant? Is it clear what sources of information were used to compile the publication? Is it clear when the information used or reported in the publication was produced? Is it balanced and unbiased? Does it provide details of additional sources of support and information? Does it refer to areas of uncertainty? Does it describe how each treatment works? Does it describe the benefits of each treatment? Does it describe the risks of each treatment? Does it describe what would happen if no treatment is used? Does it describe how the treatment choices affect overall quality of life? Is it clear that there may be more than one possible treatment choice? Does it provide support for shared decision-making? On the basis of the answers to all the above questions, rate the overall quality of the publication as a source of information about treatment choices.
HONcode [59,60]	To assess reliability and usefulness of medical information on the internet	Authority Complementarity Confidentiality Attribution Justifiability Transparency of authorship Transparency of sponsorship Honesty in advertising and editorial policy
JAMA benchmark [61]	To evaluate the reliability and accuracy of medical websites	Authorship Attribution Disclosure Currency

##### Quality Mark Indicators for Appraising Information and Advice in Online Peer Support Groups

There are specific quality mark indicators that are important and exclusive to the assessment of information and advice about health conditions in online peer support groups [44,45,48,49,51,54-56]. The source of the information and advice [45,51], currency [45,55], relevance [45,54,55], accuracy [44,51,55,56], sensibility and coherence [55,56], and the usefulness of content [51,56] were reported as key quality indicators in appraising information and advice. Many of these quality indicators mirror criteria comprising the quality appraisal tools outlined in the aforementioned section. In addition, categories such as high-quality evidence down to low-quality information [49] and “fact vs misinformation” [48] have also been used to appraise content. Moreover, web-based platform–specific metrics such as “number of posts and reposts,” “number of followers,” “likes,” and “author profile” have also been used in assessing the quality of content [45].

##### Use of Artificial Intelligence

In total, 4 (29%) studies [48,52-54] used artificial intelligence to identify and extract relevant information when assessing the quality of health information and advice. Forms of machine learning including natural language processing [52] and topic modeling [53], which is a field of study in artificial intelligence that attempts to extract general concepts from large datasets in the form of an algorithm [62], have been used in identifying specific concepts of misinformation around certain health conditions in online peer support groups [48,52-54].

## Discussion

### Summary of Evidence in Context of Wider Literature

The objective of this scoping review was to gain insight into the quality of information and advice in online peer support groups about a range of health conditions, and to review the available tools used to quality-assess such information and advice. This scoping review has identified that the quality of information and advice in online peer support groups varies according to the condition and to the degree of health care expert moderation present. While there was some evidence of good quality information and advice being exchanged [50,54-56], there was more evidence of misinformation and of low-quality information and advice, particularly around long-term and life-threatening conditions [44,45,49,51], which is also consistent with the wider literature [63,64]. There were varying degrees of misinformation about non–life-threatening conditions [48,50,55,57], viral infections [47,51,53,54,56], and mental health conditions [51,52]. In the event of a public health concern where there is uncertainty about the disease, obtaining accurate information can be extremely difficult [54].

Among platforms hosting health-related online peer support groups, Facebook presents the most misinformation about noncommunicable diseases and treatments, which may be due to there being more space in posts to describe experiences with disease symptoms and treatments [51]. X, on the other hand, appears to exhibit more misinformation about vaccines and drugs [51,52], which may be because of the natural dynamism of the platform that facilitates an ideal medium for discussions around different political or ideological perspectives [51]. In addition, the use of algorithms by web-based platforms such as Facebook and X that place content on users’ feeds that match their web-based search–related behaviors and engagement with other posts may also indicate that those whose beliefs align with misinformation are more likely to retransmit such content [23]. Effortless architecture constructed by web-based platforms, such as the one-touch forward option on WhatsApp that enables users to forward messages simultaneously to multiple users, may also explain the prevalence of misinformation on the web [65].

Platforms such as Facebook and X do not typically monitor misinformation, which may be detrimental for the continued spread of misinformation about various health conditions on such platforms. However, in the event of a public health concern such as during the COVID-19 pandemic, platforms including Facebook, X, Reddit, Instagram, and WhatsApp introduced measures to mitigate misinformation, such as placing warning labels on content about the pandemic, removing content, and banning accounts [66]. This suggests that such interventions are possible, although they are not rolled out routinely and thus may not be prioritized by platforms. The extent of the problem of misinformation in online peer support groups can alternatively be reduced by attempts from fellow online peer support group users in addressing and correcting misinformation [45,53,56]. Current moderation solutions for misinformation rely heavily on reporting mechanisms and human-led moderation, though post-by-post moderation based on individual users has been described as unsustainable [21]. Organized public health campaigns composed of factual, evidence-based messages distributed in the online world could also tackle the problem of misinformation [51-53]. Digital literacy–focused interventions that aim to teach people strategies to recognize misinformation have demonstrated effective outcomes, as well as interventions where platforms make content classed as misinformation less visible and deincentivize the creation and sharing of such content [21].

Studies have typically involved researchers, clinical academics, and specialist clinicians who hold expertise in specific conditions to conduct quality appraisals of information and advice in online peer support groups [48-50,54-57]. This has been undertaken through comparing information and advice with evidence-based guidelines [44,48-50], scientific journal articles [50], and information provided on trusted third sector websites [49]. Quality appraisal instruments including the DISCERN instrument [58], the HONcode criteria [59,60] and the JAMA benchmark [61] have been used as a recording tool for appraising the quality of information and advice exchanged around a variety of conditions and disciplines [45], despite not being designed specifically for the online social world. While criteria for forming such tools were not instinctively developed for online peer support group content, the DISCERN instrument, in particular, has been described as being suitable to evaluate any text-based health-related information (pertaining to treatment) [17]. At present, the DISCERN instrument remains in use with more than 150 published studies having reported use of the tool in evaluating health information [17], and it is recognized as a reliable tool used by both health care professionals and patient users [67]. In this review, specific quality indicators for appraising information and advice have been outlined, of which many mirror criteria forming the quality appraisal instruments discussed in this review, such as relevance [45,54,55], sources of evidence [45,51], currency [45,55], accuracy [44,51,55,56], coherence [55,56], and the usefulness of content [51,56]. Research undertaken since conducting this review also highlights accuracy, credibility, and reliability as key quality indicators for appraising information and advice in online peer support groups [67]. This suggests that the DISCERN instrument [58], the HONcode criteria [59,60], and the JAMA benchmark [61] could be appropriate quality appraisal tools for academics and health care professionals to use as a guide for appraising the quality of information and advice in online peer support groups. On occasions, these tools have been combined to assess information and advice in online peer support groups; thus, there may also be a need for novel instruments that focus solely on online support group content that would consider the nature and architecture of such content [45]. Others academics have argued that because these tools were created to be applied across different health-related domains and are therefore called “general instruments,” they may be more appropriate to evaluate factors associated with the formality of content or the design of websites rather than to evaluate the substance of content (eg, its accuracy and completeness), which may be better evaluated by domain-specific instruments that are created by researchers for individual research and are based on medical guidelines, literature, and their expertise [68].

### Strengths and Limitations of the Review

This is the first comprehensive scoping review considering the findings of a diverse and heterogeneous pool of literature and exploring the quality of information and advice in online peer support groups about various health conditions. This scoping review was conducted in accordance with the Joanna Briggs Institute methodology [35-37]. In doing so, this scoping review has successfully identified and mapped current key concepts around the quality of information and advice about various health conditions in online peer support groups, taking a broad, inclusive approach to evidence and accommodating the literature comprising a range of diverse questions and study designs.

There are also some limitations that should be considered. First, although 6 databases were searched, it is possible that some relevant studies were not identified. Second, although the search strategy was developed with the support of a specialist librarian, there are additional search terms that could have been used, such as “chat rooms” and “computer-mediated support” [69], which were excluded from the search strategy as they yielded a considerable amount of irrelevant literature. Not including these terms may have meant that some relevant literature may have been missed. However, to alleviate these potential limitations, Google Scholar was also searched, as well as the reference lists of all the included studies. This yielded additional studies that were included in our review. Third, articles focusing only on information and advice about health conditions on video-based social media sites were excluded from this review, as this paper focused on rich written information and advice generated in online communities dedicated to peer-to-peer support, rather than including comments on videos. Therefore, it is possible that some additional data were missed from comments sections on videos. Future research could compare the quality of health-related information and advice provided on web-based video platforms to traditional online peer support groups. Finally, the second part of the objective of this review was to review the available tools used to assess the quality of information and advice in online peer support groups. While this scoping review identified and mapped the available information about such tools, the articles themselves did not provide a substantive amount of information about quality appraisal tools, which limited the extent to which it was possible to review them based the included articles in this review. Wider literature has been drawn on in the discussion of this paper.

### Recommendations for Clinical Practice and Future Research

Where patients seek information and advice in the web-based world is a matter that requires more attention and improved conversations in consultations between health care professionals and patients [44,50,51]. Health care professionals need to meticulously attend to supporting people with lower levels of health literacy [29,31] and older people who may be particularly vulnerable to experiencing confusion and anxiety generated by coming across misinformation [70,71]. To do this, health care professionals need to be better informed about what online peer support groups are, how they operate, and the benefits and harms that they can offer patients. There is a need for health care professionals to be more aware of content discussed in online peer support groups [48,52-55,58] and, furthermore, to take an active role in correcting misinformation [44,45,47,48,51]. This activity is a current gap in provision and could be recommended for future career opportunities for health care professionals who may be seeking alternative roles to traditional clinical work, in meeting the health information and support needs of the public. In addition, there may be an opportunity for health care professionals in training to complete a quality appraisal task of health information available on the web and possibly to provide advice (within their field of competence) in online peer support groups. While some degree of moderation of information and advice in online peer support groups has been proven to be helpful in correcting misinformation, the extent to which health care professionals could or should be involved in moderating content as part of their duty of care was not explored in the included articles in this review. Future research is needed to determine the acceptability of and potential methods for health care professionals moderating and contributing content in online peer support groups, from the perspectives of health care professionals and patient users of online peer support groups. In addition, the type of health care professional who would be most effective in assessing the quality of content in online peer support groups, which may include general practitioners, nurses, secondary care clinicians, or others, needs to be further explored. Moreover, future research could explore the resources used in time, training, and financing around asking health care professionals to quality appraise information and advice in online peer support groups to better understand how feasible incorporating such a task would be alongside traditional clinical duties. Future research is needed to further assess the acceptability and usability of common quality appraisal tools used in appraising information and advice in online peer support groups, such as DISCERN instrument [58], the HONcode criteria [59,60], and the JAMA benchmark [61], for health care professionals to use them as a guide in such practice. There is a need for a novel instrument tailored specifically to appraise health-related information in online peer support groups, incorporating items focusing on reliability, credibility, accuracy, currency, coherence, and usefulness as the basis of the tool.

### Conclusions

There is increasing concern regarding the quality of information and advice about health conditions exchanged in online peer support groups. While there is good quality information and advice exchanged between users, misinformation is also a problem, with some users being ill-advised about common long-term and life-threatening conditions, viral infections, and mental health conditions. The extent of the problem of misinformation can be reduced with fellow users in online peer support groups addressing and correcting misinformation and through public health campaigns organized in the online world. Health care professionals and patients need to discuss the use of online peer support groups more regularly in consultations. Furthermore, there is a need for health care professionals to play a more active role in quality-assuring health-related information and advice in online peer support groups. This could be facilitated using established quality appraisal tools such as the DISCERN instrument, the HONcode criteria, and the JAMA benchmark as a guide in appraising content. Future research is needed focusing on approaches to educating health care professionals around the benefits and harms that online peer support groups can offer patients, along with investigating the acceptability of and potential methods for health care professionals moderating and contributing content in online peer support groups.

## Appendix

Multimedia Appendix 1 Final search strategy for all included databases and sources to obtain literature exploring the quality of information and advice about health conditions in online peer support groups.

Multimedia Appendix 2 Health conditions explored and reported quality of information and advice across the 14 included articles, exploring the quality of information and advice in online peer support groups.

Multimedia Appendix 3 Web-based platforms explored across the 14 included articles exploring the quality of information and advice about health conditions in online peer support groups.

Multimedia Appendix 4 PRISMA-ScR checklist.

## Abbreviations

HONcode: Health on the Net Code of Conduct
JAMA: Journal of the American Medical Association
PCC: Population, Concept, and Context
PRISMA: Preferred Reporting Items for Systematic Reviews and Meta-Analyses
PRISMA-ScR: Preferred Reporting Items for Systematic Reviews and Meta-Analyses extension for Scoping Reviews
RQ: research question
