# Supplementary material for: Quality and Misinformation About Health Conditions in Online Peer Support Groups: Scoping Review
Source: J Med Internet Res. 2025 May 16;27:e71140. doi: 10.2196/71140 (PMC12125560; doi:10.2196/71140)
Supplement: Multimedia Appendix 1 [file jmir_v27i1e71140_app1.docx]

**Quality and misinformation about health conditions in online peer support groups: Scoping review**

Bethan M. Treadgold, Neil S. Coulson, John L. Campbell, Jeffrey Lambert, Emma Pitchforth

**Supplementary file 1: Final search strategy for all included databases and sources to obtain literature exploring the quality of information and advice about health conditions in online peer support groups.**

| **Medline (Ovid)** | **Web of Science (Clivariate)** | **Applied Social Sciences Index & Abstracts (ProQuest)** | **CINAHL (Ebscohost)** | **ProQuest Dissertation and Theses** | **Google Scholar** |
| --- | --- | --- | --- | --- | --- |
| 1. *Self-Help Groups/ or online support group*.mp. or *Social Support/  2. (online communit* or social network*).mp.  3. *Social Media/ or online discussion forum*.mp.  4. *Quality Control/ or *Quality Assurance, Health Care/ or quality assessment.mp.  5. medical accuracy.mp.  6. quality appraisal.mp.  7. (health information or information).mp. or *Consumer Health Information/  8. advice.mp.  9. 1 or 2 or 3  10. 4 or 5 or 6  11. 7 or 8  12. 9 and 10 and 11 | (TI=(online support group*) OR TI=(online communit*) OR TI=(discussion forum*) OR TI=(social media*) OR TI=(social network*))  AND  (TI=(quality) OR TI=(assessment*) OR TI=(evaluat*) OR TI=(accuracy) OR TI=(appraisal))  AND  (TI=(information) OR TI=(advice)) | (noft(online support group*) OR noft(online communit*) OR noft(discussion forum*) OR noft (social network*) OR noft(social media*))  AND  (noft(quality assessment) OR noft(accuracy) OR noft(appraisal) OR noft(evaluat*))  AND  (noft(information) OR noft(advice)) | S1. TI online support group* OR TI online communit* OR TI discussion forum* OR TI social media* OR TI social network  S2. TI quality asess* OR TI quality OR TI assess* OR TI accuracy OR TI apprais*  S3. TI information OR TI advice  S4. (TI information OR TI advice) AND (S1 AND S2 AND S3)  S5. (((TI information OR TI advice) AND (S2 AND S3 AND S4)) AND (S1 AND S2 AND S3)) AND (S2 AND S3) | (title(online support group*) OR title(online communit*) OR title(discussion forum*) OR title(social network*) OR title(social media*))  AND  (title(quality assessment) OR title(accuracy) OR title(appraisal) OR title(evaluat*))  AND  (title(information) OR title(advice)) | (online support group* OR online communit* OR discussion forum* OR social network* OR social media*)  AND  (quality assess* OR accuracy OR apprais* OR evaluat*)  AND  (information OR advice) |
