# Supplementary material for: Quality and Misinformation About Health Conditions in Online Peer Support Groups: Scoping Review
Source: J Med Internet Res. 2025 May 16;27:e71140. doi: 10.2196/71140 (PMC12125560; doi:10.2196/71140)
Supplement: Multimedia Appendix 3 [file jmir_v27i1e71140_app3.docx]

**Quality and misinformation about health conditions in online peer support groups: Scoping review**

Bethan M. Treadgold, Neil S. Coulson, John L. Campbell, Jeffrey Lambert, Emma Pitchforth

**Supplementary file 3: Web-based platforms explored across the 14 included articles exploring the quality of information and advice about health conditions in online peer support groups.**

| **Online platform** | **Description** | **References** |
| --- | --- | --- |
| Facebook | Social media and social networking service where users can share information via posts through their own Facebook profile, within public, private, and secret groups, and on pages. | [44-45,48-49, 55, 57] |
| X (formerly Twitter) | Social media and social networking service where users can share information via posts through their own Twitter profile, publicly or privately. | [44-45, 49-50, 54-55] |
| Reddit | Social media and social networking service where users can share information via posts through their own Reddit profile, and within discussion forums, either publicly or privately. | [44-46, 50-51, 56] |
| Instagram | Social media and social networking service where users can share photos and videos via posts through their own Instagram profile, either publicly or privately. | [44-45] |
| Whatsapp | Instant messaging app where users can send messages to others individually or via a group, through mobile phone numbers. | [45,49] |
| Other (Wechat, Weibo, myspace, telegram, interia, parler, VK, Drugs-Forum, The Naver Q&A forum, patient, mumsnet, The International Glaucoma Association forum, Patient-Opinion) | Mixture of less commonly cited social media, social networking, and online discussion forum sites. | [45,49,55] |

**References**

1. Wollmann K, van der Keylen P, Tomandl J, Meerpohl JJ, Sofroniou M, Maun A, et al. The information needs of internet users and their requirements for online health information—A scoping review of qualitative and quantitative studies. Patient Education and Counseling. 2021;104(8):1904-32.

2. Luo A, Qin L, Yuan Y, Yang Z, Liu F, Huang P, et al. The effect of online health information seeking on physician-patient relationships: systematic review. Journal of Medical Internet Research. 2022;24(2):e23354.

3. Robinson TN, Patrick K, Eng TR, Gustafson D. An evidence-based approach to interactive health communication: A challenge to medicine in the information age. Jama. 1998;280(14):1264-9.

4. Le LH, Hoang PA, Pham HC. Sharing health information across online platforms: A systematic review. Health Communication. 2023;38(8):1550-62.

5.. Tausczik Y, Huang X. Knowledge generation and sharing in online communities: Current trends and future directions. Current opinion in psychology. 2020;36:60-4.

6. Berkanish P, Pan S, Viola A, Rademaker Q, Devine KA. Technology-based peer support interventions for adolescents with chronic illness: a systematic review. Journal of clinical psychology in medical settings. 2022;29(4):911-42.

7. Frey E, Bonfiglioli C, Brunner M, Frawley J. Parents’ use of social media as a health information source for their children: A scoping review. Academic pediatrics. 2022;22(4):526-39.

8. Newman K, Wang AH, Wang AZY, Hanna D. The role of internet-based digital tools in reducing social isolation and addressing support needs among informal caregivers: a scoping review. BMC Public Health. 2019;19:1-12.

9. Sweet KS, LeBlanc JK, Stough LM, Sweany NW. Community building and knowledge sharing by individuals with disabilities using social media. Journal of computer assisted learning. 2020;36(1):1-11.

10. Johansson V, Islind AS, Lindroth T, Angenete E, Gellerstedt M. Online communities as a driver for patient empowerment: systematic review. Journal of medical Internet research. 2021;23(2):e19910.

11. Hwang EH, Singh PV, Argote L. Knowledge sharing in online communities: Learning to cross geographic and hierarchical boundaries. Organization Science. 2015;26(6):1593-611.

12. Allen C, Vassilev I, Kennedy A, Rogers A. Long-term condition self-management support in online communities: a meta-synthesis of qualitative papers. Journal of medical Internet research. 2016;18(3):e61.

13. Ziebland S, Wyke S. Health and illness in a connected world: how might sharing experiences on the internet affect people's health? The Milbank Quarterly. 2012;90(2):219-49.

14. Treadgold BM, Teasdale E, Muller I, Roberts A, Coulson N, Santer M. Parents and Carers’ experiences of seeking health information and support Online for long-term physical childhood conditions: A systematic review and thematic synthesis of qualitative research. BMJ open. 2020;10(12):e042139.

15. Griffiths F, Dobermann T, Cave JA, Thorogood M, Johnson S, Salamatian K, et al. The impact of online social networks on health and health systems: a scoping review and case studies. Policy & internet. 2015;7(4):473-96.

16. Organization WH. Toolkit for tackling misinformation on noncommunicable disease: forum for tackling misinformation on health and NCDs. 2022 [Available from: https://www.who.int/europe/publications/i/item/WHO-EURO-2022-6260-46025-66542.

17. Keselman A, Arnott Smith C, Murcko AC, Kaufman DR. Evaluating the quality of health information in a changing digital ecosystem. Journal of medical Internet research. 2019;21(2):e11129.

18. Daraz L, Morrow AS, Ponce OJ, Beuschel B, Farah MH, Katabi A, et al. Can patients trust online health information? A meta-narrative systematic review addressing the quality of health information on the internet. Journal of general internal medicine. 2019;34:1884-91.

19. Wagner T, Howe CJ, Lewis B, Adame T. Is Your WebLitLegit? Finding safe and good health information on the Internet. HLRP: Health Literacy Research and Practice. 2022;6(2):e151-e8.

20. Al-Jefri M, Evans R, Uchyigit G, Ghezzi P. What is health information quality? Ethical dimension and perception by users. Frontiers in medicine. 2018 Sep 20;5:260.

21. Johansson P, Enock F, Hale S, Vidgen B, Bereskin C, Margetts H, Bright J. How can we combat online misinformation? A systematic overview of current interventions and their efficacy. arXiv preprint arXiv:2212.11864. 2022 Dec 22.

22. Borges do Nascimento IJ, Pizarro AB, Almeida JM, Azzopardi-Muscat N, Gonçalves MA, Björklund M, Novillo-Ortiz D. Infodemics and health misinformation: a systematic review of reviews. Bull World Health Organ. 2022 Sep 1;100(9):544-56123.

23. Muhammed T S, Mathew SK. The disaster of misinformation: a review of research in social media. Int J Data Sci Anal. 2022;13(4):271-285. doi: 10.1007/s41060-022-00311-6. Epub 2022 Feb 15. PMID: 35194559; PMCID: PMC8853081.

24. Chou W-YS, Oh A, Klein WM. Addressing health-related misinformation on social media. Jama. 2018;320(23):2417-8.

25. Hajli MN, Sims J, Featherman M, Love PE. Credibility of information in online communities. Journal of Strategic Marketing. 2015;23(3):238-53.

26. Zhao Y, Da J, Yan J. Detecting health misinformation in online health communities: Incorporating behavioral features into machine learning based approaches. Information Processing & Management. 2021;58(1):102390.

27. Ma TJ, Atkin D. User generated content and credibility evaluation of online health information: a meta analytic study. Telematics and Informatics. 2017;34(5):472-86.

28. Adams SA. Revisiting the online health information reliability debate in the wake of “web 2.0”: an inter-disciplinary literature and website review. International journal of medical informatics. 2010;79(6):391-400.

29. Petrič G, Cugmas M, Petrič R, Atanasova S. The quality of informational social support in online health communities: a content analysis of cancer-related discussions. Digital health. 2023;9:20552076231155681.

30. Akbolat M, Amarat M, Ünal Ö, Şantaş G. A survey of health information seeking by cancer patients indicates some problems over medical explanations and terminology. Health Information & Libraries Journal. 2023;40(1):29-41.

31. Sudau F, Friede T, Grabowski J, Koschack J, Makedonski P, Himmel W. Sources of information and behavioral patterns in online health forums: observational study. Journal of medical Internet research. 2014;16(1):e10.

32. Coleman, A.: Hundreds dead’ because of Covid-19 misinformation—BBC News. BBC News (2020).

33. Peterson J, Pearce PF, Ferguson LA, Langford CA. Understanding scoping reviews: Definition, purpose, and process. Journal of the American Association of Nurse Practitioners. 2017;29(1):12-6.

34. Anderson S, Allen P, Peckham S, Goodwin N. Asking the right questions: scoping studies in the commissioning of research on the organisation and delivery of health services. Health research policy and systems. 2008;6:1-12.

35. Tricco AC, Lillie E, Zarin W, O’brien K, Colquhoun H, Kastner M, et al. A scoping review on the conduct and reporting of scoping reviews. BMC medical research methodology. 2016;16:1-10.

36. Pollock D, Peters MD, Khalil H, McInerney P, Alexander L, Tricco AC, et al. Recommendations for the extraction, analysis, and presentation of results in scoping reviews. JBI evidence synthesis. 2023;21(3):520-32.

37. Peters MD, Marnie C, Tricco AC, Pollock D, Munn Z, Alexander L, et al. Updated methodological guidance for the conduct of scoping reviews. JBI evidence synthesis. 2020;18(10):2119-26.

38. Tricco AC, Lillie E, Zarin W, O'Brien KK, Colquhoun H, Levac D, Moher D, Peters MD, Horsley T, Weeks L, Hempel S. PRISMA extension for scoping reviews (PRISMA-ScR): checklist and explanation. Annals of internal medicine. 2018 Oct 2;169(7):467-73.

39. Treadgold B, Coulson N, Campbell J, Lambert J, Pitchforth E. Quality of health-related information and advice in online support groups: Protocol for a scoping review. Open Science Framework. 2024. [Available from: https://doi.org/10.17605/OSF.IO/U9XFY].

40. Covidence systematic review software VHI. 2024. Melbourne, Australia [Available from: http://www.covidence.org/.

41. Haddaway NR, Collins AM, Coughlin D, Kirk S. The role of Google Scholar in evidence reviews and its applicability to grey literature searching. PloS one. 2015;10(9):e0138237.

42. NVivo qualitative data analysis computer software 14 L. Colorado, United States of America. 2024 [Available from: https://lumivero.com/products/nvivo/].

43. Page MJ, McKenzie JE, Bossuyt PM, Boutron I, Hoffmann TC, Mulrow CD, et al. The PRISMA 2020 statement: an updated guideline for reporting systematic reviews. Bmj. 2021;372.

44. Slick N, Bodas P, Badawy SM, Wildman B. Accuracy of online medical information: The case of social media in sickle cell disease. Pediatric Hematology and Oncology. 2023;40(2):99-107.

45. Afful-Dadzie E, Afful-Dadzie A, Egala SB. Social media in health communication: A literature review of information quality. Health Information Management Journal. 2023;52(1):3-17.

46. Ulep AJ, Deshpande AK, Beukes EW, Placette A, Manchaiah V. Social media use in hearing loss, tinnitus, and vestibular disorders: A systematic review. American Journal of Audiology. 2022;31(3S):1019-42.

47. Skafle I, Nordahl-Hansen A, Quintana DS, Wynn R, Gabarron E. Misinformation about COVID-19 vaccines on social media: rapid review. Journal of medical Internet research. 2022;24(8):e37367.

48. Pollack CC, Emond JA, O'Malley AJ, Byrd A, Green P, Miller KE, et al. Characterizing the prevalence of obesity misinformation, factual content, stigma, and positivity on the social media platform Reddit between 2011 and 2019: Infodemiology study. Journal of Medical Internet Research. 2022;24(12):e36729.

49. Farnood A, Johnston B, Mair FS. An analysis of the diagnostic accuracy and peer‐to‐peer health information provided on online health forums for heart failure. Journal of Advanced Nursing. 2022;78(1):187-200.

50. Towne J, Suliman Y, Russell KA, Stuparich MA, Nahas S, Behbehani S. Health information in the era of social media: An analysis of the nature and accuracy of posts made by public Facebook pages for patients with endometriosis. Journal of minimally invasive gynecology. 2021;28(9):1637-42.

51. Suarez-Lledo V, Alvarez-Galvez J. Prevalence of health misinformation on social media: systematic review. Journal of medical Internet research. 2021;23(1):e17187.

52. ElSherief M, Sumner SA, Jones CM, Law RK, Kacha-Ochana A, Shieber L, et al. Characterizing and identifying the prevalence of web-based misinformation relating to medication for opioid use disorder: machine learning approach. Journal of medical Internet research. 2021;23(12):e30753.

53. Du J, Preston S, Sun H, Shegog R, Cunningham R, Boom J, et al. Using machine learning–based approaches for the detection and classification of human papillomavirus vaccine misinformation: Infodemiology study of reddit discussions. Journal of Medical Internet Research. 2021;23(8):e26478.

54. Jo W, Lee J, Park J, Kim Y. Online information exchange and anxiety spread in the early stage of the novel coronavirus (COVID-19) outbreak in South Korea: structural topic model and network analysis. Journal of medical Internet research. 2020;22(6):e19455.

55. Sepehripour S, McDermott AL, Lloyd MS. Microtia and social media: patient versus physician perspective of quality of information. Journal of Craniofacial Surgery. 2017;28(3):643-5.

56. Cole J, Watkins C, Kleine D. Health advice from internet discussion forums: how bad is dangerous? Journal of medical Internet research. 2016;18(1):e4.

57. McGregor F, Somner JE, Bourne RR, Munn‐Giddings C, Shah P, Cross V. Social media use by patients with glaucoma: what can we learn? Ophthalmic and Physiological Optics. 2014;34(1):46-52.

58. Charnock D, Shepperd S, Needham G, Gann R. DISCERN: an instrument for judging the quality of written consumer health information on treatment choices. Journal of Epidemiology & Community Health. 1999;53(2):105-11.

59. Boyer C, Frossard C, Gaudinat A, Hanbury A, Falquetd G. How to sort trustworthy health online information? Improvements of the automated detection of HONcode criteria. Procedia computer science. 2017;121:940-9.

60. Boyer C, Selby M, Scherrer J-R, Appel RD. The health on the net code of conduct for medical and health websites. Computers in biology and medicine. 1998;28(5):603-10.

61. Silberg WM, Lundberg GD, Musacchio RA. Assessing, controlling, and assuring the quality of medical information on the Internet: Caveant lector et viewor—Let the reader and viewer beware. Jama. 1997;277(15):1244-5.

62. Doupe P, Faghmous J, Basu S. Machine learning for health services researchers. Value in Health. 2019;22(7):808-15.

63. Alipour S, Nikooei S, Hosseinpour R, Yavari Barhaghtalab MJ. Evaluation of the quality and accuracy of breast cancer knowledge among persian language websites. BMC Health Serv Res. 2022;22(1):1560.

64. Reidy C, Klonoff DC, Barnard-Kelly KD. Supporting good intentions with good evidence: how to increase the benefits of diabetes social media. J Diabetes Sci Technol. 2019;13(5):974–8.

65. Bapaye JA, Bapaye HA. Demographic Factors Influencing the Impact of Coronavirus-Related Misinformation on WhatsApp: Cross-sectional Questionnaire Study. JMIR Public Health Surveill. 2021 Jan 30;7(1):e19858. doi: 10.2196/19858. PMID: 33444152; PMCID: PMC7850780.

66. Krishnan, N., Gu, J., Tromble, R., & Abroms, L. C. (2021). Research note: Examining how various social media platforms have responded to COVID-19 misinformation. Harvard Kennedy School (HKS) Misinformation Review. https://doi.org/10.37016/mr-2020-85

67. Ghalavand, H., Nabiolahi, A. Exploring online health information quality criteria on social media: a mixed method approach. BMC Health Serv Res 24, 1311 (2024). https://doi.org/10.1186/s12913-024-11838-8

68. Zhang Y, Sun Y, Xie B. Quality of health information for consumers on the web: a systematic review of indicators, criteria, tools, and evaluation results. Journal of the Association for Information Science and Technology. 2015 Oct;66(10):2071-84.

69. Coulson NS, Buchanan H. The role of online support groups in helping individuals affected by HIV and AIDS: scoping review of the literature. Journal of Medical Internet Research. 2022;24(7):e27648.

70. Vivion, M., Reid, V., Dubé, E. et al. How older adults manage misinformation and information overload - A qualitative study. BMC Public Health 24, 871 (2024). https://doi.org/10.1186/s12889-024-18335-x

71. Scherer, L. D., & Pennycook, G. (2020). Who Is Susceptible to Online Health Misinformation?. American journal of public health, 110(S3), S276–S277. https://doi.org/10.2105/AJPH.2020.305908
